# Problematic behaviors at mealtimes and the nutritional status of Brazilian children with Autism Spectrum Disorder

**DOI:** 10.3389/fpubh.2024.1392478

**Published:** 2024-10-14

**Authors:** Rita de Cassia Santos Soares, Flávia Galvão Cândido, Mariana De Santis Filgueiras, Carla de Oliveira Barbora Rosa, Juliana Farias de Novaes, Raquel Maria Amaral Araujo

**Affiliations:** Department of Nutrition and Health (DNS), Federal University of Viçosa (UFV), Minas Gerais, Brazil

**Keywords:** food consumption, eating behavior, nutritional status, Autism Spectrum Disorder, food

## Abstract

**Objective:**

The aim of this study was to explore problematic behaviors and their association with the food consumption and nutritional status of children with Autism Spectrum Disorder (ASD).

**Methods:**

This is a cross-sectional study which enrolled 90 Brazilian children (aged 2–10 years) with ASD. A sociodemographic questionnaire, the ASD Eating Behavior Assessment Scale, and the 24-h recall were used. Age, height, total body weight, and Body Mass Index (BMI) were accessed to estimate nutritional status (BMI/Age). Consumption of fruits, vegetables, total calories, macro and micronutrients (calcium, iron, zinc, omega 3 and omega 6), and Body Mass Index (BMI) were assessed. The regression models were adjusted for covariates (prevalence ratio – PR, α =  0.05).

**Results:**

All studied children presented problematic behaviors at mealtimes, with a predominance of food selectivity (57.8%), followed by changes in mealtime skills (34.4%), changes in masticatory motor skills (25.6%), oppositional eating behaviors (20.4%), and rigid eating behaviors (20.0%). Food selectivity was associated with excess body weight (PR: 1.94; 95% CI: 1.08–3.51) and absence of vegetable consumption (PR: 0.61; 95% CI: 0.46–0.81). Rigid eating behaviors was associated with low consumption of fiber (PR: 1.42; 95% CI: 1.15–1.75) and zinc (PR: 2.15; 95% CI: 1.33–3.45), and with excessive caloric (PR: 1.14; 95% CI: 1.02–1.29) and lower calcium intake (PR: 0.52; 95% CI: 0.29–0.91).

**Conclusion:**

Selectivity and rigidity behaviors have different relationships with food consumption and are associated with a higher prevalence of inadequacies, which may lead to nutritional risks for children. Further studies may investigate the influence of problematic behaviors and excess body weight in ASD.

## Introduction

Autism Spectrum Disorder (ASD) is a common neurodevelopmental disorder, characterized by atypical patterns of activities and behaviors (1). It is estimated that one in every 36 eight-year-old children in the United States is diagnosed with ASD (2). In Brazil, there is still no official data on the prevalence of ASD at the national level. Although the government requested the inclusion of this information, the data is still under investigation (2) and prevalence of ASD in Brazilians remains restricted to studies with a less comprehensive sample (3). Nevertheless, the Ambulatory Information System shows that, in 2021, Brazil carried out 9.6 million outpatient visits for people with ASD, 4.1 million of which were for children up to 9 years of age (2).

The nutritional management of children with ASD represents one of the challenges of health care (4), given that problematic behaviors at mealtimes are more frequent in this group compared to those without ASD (5). Therefore, interest has grown in understanding the relationships between these behaviors and the quality of food and nutritional status of people with ASD (4, 5). It have been postulated that diet of children with ASD can be extremely restricted, leading to a risk of nutritional deficiencies and gastrointestinal disorders (1, 4, 5). On the other hand, prevalence of excess body weight is higher in children with ASD than those in the general population (4–6), although a higher proportion of underweight is also observed (4). A meta-analysis (7) to examine the association between obesity, overweight and ASD, demonstrate a positive association between obesity and ASD, despite no association between overweight and ASD. Considering the inconsistence of such associations (7) and that there are a variety of problematic behaviors at mealtimes in ASD (1), it can be hypothesized that the deviations in the nutritional status could be partially due to individual differences in the presentation of problematic behaviors, since it could affect food consumption. Thus, investigating the influence of specific problematic behaviors at mealtimes on food consumption and nutritional status could favors a better understanding of such relationships and to subside more appropriated nutritional interventions in children with ASS.

Among the problematic behaviors at mealtimes in ASD, the food selectivity, characterized by restricted eating patterns, food refusal, and consumption of a small variety of foods (4, 8, 9), is the most common (10) and is related to tactile and oral sensibility to the texture of food (11), resulting in consumption of a small variety of vegetables and fruits (12) and preference for liquid texture (10). On the other hand, rigid eating behaviors is characterized by the insistence on “sameness,” difficulties with changes in routine or environment, ritualistic behaviors and limited interests (13).

Problematic behaviors at mealtimes in ASD are related to dietary and nutritional parameters, influencing the quantity and quality of foods consumed, especially vegetables, fruits, and milk, compromising the intake of calories and various nutrients associated with growth and cognitive development, such as iron, calcium, zinc, polyunsaturated fatty acids (PUFA) of the omega 3 and omega 6 series, and fibers (4, 5, 8). The most studied relationships concern food selectivity behavior, however the results are conflicting (14), possibly influenced by the different ways of characterizing food selectivity (15) and to methodological inaccuracy (14). Considering that oral texture sensibility and rigidity are independent predictors of food consumption (16), investigating the relationships of food selectivity and rigid eating behaviors with dietary and nutritional parameters can favor to better understanding the nutritional risks related to each behavior. Therefore, the present study aimed to elucidate the relationships between problematic behaviors at mealtimes, food consumption, and the nutritional status of children with ASD.

## Methods

### Study design

This is a cross-sectional study carried out with all children (aged 2–10 years), of both sexes, diagnosed with ASD according to the International Classification of Diseases ICD-11 (17), attending a reference institution for interdisciplinary health care of children with any type of neurodevelopmental disorders of a microregion in the state of Minas Gerais, in Brazil, from August to December 2022. Of the 99 children with ASD, six were not included due to their mothers’ refusal and three dropped out of the study, resulting in 90 children and a loss percentage of 9.1% (*n* = 9).

The power of the study was calculated using the online OpenEpi Software, adopting the frequency of consumption of vegetables and legumes among children with the highest score in mealtime skills (51.9%) and those with the lowest score (92.1%), considering a 95% confidence interval. The power of the study obtained was 98.1%. Exclusion criteria covered the presence of a disease that requires changes in diet, such as ketogenic diets, autism secondary to genetic syndromes, or those whose mothers refused to participate in the research. The data were collected by previously trained researchers and the entire team was trained by the main researcher, who has long experience in nutritional care of children with ASD, and is therefore knowledgeable about the specificities of care for this group. The study was approved by the Research Ethics Committee of the Federal University of Viçosa (n° 56933622.3.0000.5153). All participant’s mothers provided informed consent.

### Data collection

A semi-structured questionnaire was applied to provide sociodemographic information about the mother and child, and about feeding in early childhood. Birth data and CID 1130 were obtained from electronic medical records. The socioeconomic classification was based on the criteria of the Brazilian Association of Research Companies (18). The Brazilian Economic Classification Criterion is a system used to categorize the Brazilian population into different economic classes.

In Brazil, social classes are traditionally defined based on monthly household income measured in minimum wages: Class A includes families with more than 20 minimum wages, having high purchasing power and access to luxury goods and services; Class B falls between 10 and 20 minimum wages, with good access to private education and healthcare; Class C, considered the middle class, ranges from 4 to 10 minimum wages, with moderate consumption and access to essential goods; Class D has between 2 and 4 minimum wages, focusing on basic needs; and Class E, with less than 2 minimum wages, faces limited access to goods and services and often relies on social assistance programs (18). The following variables were categorized: economic class (B1/B2/C1 and C2/DE), skin color (white and brown/black), maternal education (1 – illiterate or unfinished elementary I; 2 – finished elementary I or unfinished elementary II; 3 – finished elementary II or unfinished high school education; 4 – finished high school or unfinished higher education; 5 – finished higher education), maternal work (mothers who work outside the home and mothers who do not work) and timely introduction of complementary feeding (before and after 6 months of the child’s life) (19).

### Nutritional status

Weight and height measurements followed the recommended methodology (20), and their values were converted into the Body Mass Index for Age (BMI/Age). An electronic scale certified by INMETRO was used for weighing, with a maximum capacity of 150 kg and precision of 0.1 kg. Height was measured in the standing position with the aid of a GPM^®^ portable anthropometer, with an accuracy of 0.1 cm. BMI/Age was used to describe nutritional status through the WHO-Anthro (2011) (21) and WHO-Anthro Plus (2011) (21) programs, generating Z-score values used for the description and analysis of the studied population. The growth curves defined by the WHO (22, 23) were adopted. BMI/Age was categorized into severe thinness/thinness, normal weight, overweight, obesity/severe obesity. For association analyses, the BMI/Age variable was dichotomized into: without excess body weight (severe thinness, thinness, and normal weight) and with excess body weight (overweight, obesity, and severe obesity).

### Assessment of food consumption

Assessment of food consumption was performed by 24-h recall (24HR) applied on three non-consecutive days, one on the weekend. Typing and analyzes were performed in duplicate. The DietPRO^®^ (24) software was used to obtain the caloric, macro and micronutrient composition from the tables: Brazilian Table of Food Composition (TACO) (25); DietPRO^®^ Software Table and National Nutrient Database for Standard Reference (USDA) (26), in sequence. The Dietary Reference Intake Recommendations (27) were used to analyze the adequacy of total calories, protein, iron, calcium, zinc, fiber, polyunsaturated fatty acids omega 3 and omega 6, which were variables used in the analyses of association with problematic mealtime behaviors. Inadequacy of nutrient consumption was defined for values below the RDA for the corresponding age groups (27). Calorie inadequacy was identified for values both below and above those recommended for the corresponding age groups (27). The consumption of fruits and vegetables was assessed by checking their presence or absence in meals, as children with food selectivity tend to reject these foods, not considering the consumption of fruit and vegetable juice.

### Eating behavior

The study of the eating behavior of children with ASD was based on the ASD Eating Behavior Assessment Scale, developed in Brazil (28), which identifies seven dimensions of eating behavior that are altered, namely: (1) masticatory motor skills; (2) food selectivity; (3) mealtime skills; (4) inappropriate mealtime behaviors; (5) rigid eating behaviors; (6) oppositional eating behavior; and (7) food allergies and intolerances. The questionnaire comprised 26 items distributed among the seven dimensions and a 5-point Likert scale, ranging from never to always. The average scores were calculated for each dimension and then the population was divided into two groups: those with scores above and below the average for each dimension.

### Statistical analysis

Statistical analyses were performed using STATA^®^, version 14.0. Numerical variables were expressed as mean and standard deviation, and categorical variables as absolute (n) and relative frequency (%). Pearson’s chi-square and Fisher’s exact tests were used to verify the association between two categorical variables. Fisher’s exact test was used to analyze statistical differences between categorical variables when more than 20% of the cells had an expected count lower than five. These tests were used to evaluate the association of the Eating Behavior Scale with sociodemographic and maternal characteristics, timely introduction of complementary foods, excess weight, and food consumption. Poisson regression models with robust variance were used to calculate the prevalence ratio (PR) and their respective 95% confidence intervals (95%CI). The association between the items of the Eating Behavior Scale (exposure variables) with excess body weight and food consumption variables (outcome variables) was assessed. All models were adjusted for potential confounding factors, selected through a literature review. The variable was adjusted for age, sex, skin color, maternal profession, economic class and medicaments. In these analyses, dimensions four and seven were excluded due to their low occurrence. The adequacy of the models was assessed using the Hosmer & Lemeshow goodness-of-it test. For all hypothesis tests, a significance level of 5% was used.

## Results

The majority of children were white (46.7%), belonged to socioeconomic classes C, D, and E (68.9%), were eutrophic (56.7%), and had timely introduction of complementary feeding (75.6%). The majority of mothers had finished high school or above (66%) and worked outside the home (56.7%) (Table 1).

**Table 1:** Characterization of Brazilian children (2–10 years) with autism spectrum disorder, Viçosa, Brazil (2022).

Characteristics	*n* (%)
Sex	
Feminine	21 (23.3)
Masculine	69 (76.7)
Skin color	
White	42 (46.7)
Brown	33 (36.7)
Black	15 (16.7)
Socioeconomic class	
B1	7 (7.8)
B2	21 (23.3)
C1	15 (16.7)
C2	21 (23.3)
DE	26 (28.9)
Timely introduction of complementary feeding	
Yes	68 (75.6)
No	22 (24.4)
BMI/Age	
Accentuated thinness/Thinness	2 (2.2)
Eutrophy	51 (56.7)
Overweight	19 (21.1)
Obesity/Severe obesity	18 (20.0)
Maternal education	
Illiterate/Unfinished elementary school	1 (1.1)
Finished elementary I/Unfinished elementary II	9 (10.0)
Finished elementary II/Unfinished high school	14 (15.6)
Finished high school/Unfinished higher education	48 (53.3)
Finished higher education	18 (20.0)
Maternal profession	
Yes	51 (56.7)
No	39 (43.3)
Medication use	
Yes	56 (62.2)
No	34 (37.8)

BMI/Age, Body Mass Index for age. Economic class and maternal education were classified according to ABEP (18). Three mothers who declared themselves students were classified as “no” for maternal profession. Medicines: Risperidone, methylphenidate, carnabidiol, sodium valproate, fluoxetine hydrochloride, levomepromazine hydrochloride, amitriptyline hydrochloride lisdexamfetamine, quetiapine, paroxetine, aripiprazole, melatonin.

### Problematic mealtime behaviors

**Figure 1:**
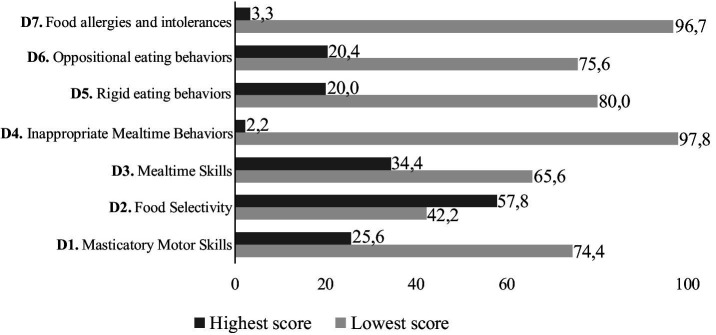
Frequency of problematic mealtime behaviors of Brazilian children (2–10 years) with autism spectrum disorder (*n* = 90), Viçosa, Brazil (2022). Reference values per dimension (D), maximum score; cut-off point: D1 (16; 8); D2 (12; 6); D3 (20; 10); D4 (8; 4); D5 (24; 12); D6 (12; 6); D7 (12; 6).

All children showed at least one problematic mealtime eating behavior, and all investigated behaviors were detected in the studied group. Food selectivity was the most common, present in 57.8% of children, followed by changes in mealtime skills (34.4%), changes in masticatory motor skills (25.6%), oppositional eating behavior (20.4%), and rigid eating behavior (20.0%). The frequency of children with food allergies and intolerances, as well as inappropriate mealtime behaviors, was low in the studied group (3.3 and 2.2%, respectively) (Figure 1).

### Associations of problematic mealtime behaviors with nutritional status and food consumption

Excess body weight was present in 41.1% of children, which overweight and obesity presenting rates of 21.1 and 20.0%, respectively.

Food selectivity was associated with not consuming vegetables (*p* = 0.001) and with excess body weight (*p* = 0.045). Rigid eating behaviors ware associated with calcium adequacy (*p* = 0.005), lower zinc (*p* = 0.001) and fiber consumption (*p* = 0.035) (Supplementary Table 1). The final adjusted model of the Poisson regression analysis (Table 2) demonstrates that those children with higher scores of food selectivity had higher prevalence of excess body weight (PR: 1.94; 95% CI: 1.08–3.51), and of absence of vegetable consumption (PR: 0.61; 95% CI: 0.46–0.81). Furthermore, higher scores for rigid eating behaviors was positively associated with excessive caloric intake (PR: 1.14; 95% CI: 1.02–1.29) and with lower fiber (PR: 1.42; 95% CI: 1.15–1.75) and zinc intake (PR: 2.15; 95% CI: 1.33–3.45). Higher scores for rigid eating behaviors was also associated with lower prevalence of inadequate calcium intake (PR: 0.52; 95% CI: 0.29–0.91).

**Table 2:** Association between problematic behaviors at mealtimes with inadequacies in food consumption and nutritional status in Brazilian children (2–10 years) with autism spectrum disorder (*n* = 90), Viçosa, Brazil (2022).

Characteristics	Iron PR (CI95%)	Calcium PR (CI95%)	Zinc PR (CI95%)	Omega3 PR (CI95%)	Omega6 PR (CI95%)
D1 < 8 ≥ 8P	0.50 (0.17–1.47) 0.209	0.86 (0.60–1.22) 0.397	1.0 (0.59–1.67) 0.989	0.74 (0.26–2.06) 0.561	0.78 (0.26–2.31) 0.653
D < 6 ≥ 6 P	0.67 (0.33–1.38) 0.279	1.00 (0.75–1.33) 0.991	0.88 (0.54–1.44) 0.612	0.70 (0.31–1.55) 0.374	1.45 (0.70–2.98) 0.315
D3 < 10 ≥ 10P	1.17 (0.58–2.33) 0.663	0.98 (0.75–1.29) 0.881	0.97 (0.57–1.67) 0.924	1.65 (0.80–3.41) 0.173	1.05 (0.46–2.38) 0.905
D5 < 12 ≥ 12P	1.69 (0.72–3.97) 0.230	0.52 (0.29–0.91) 0.021*	2.15 (1.33–3.45) 0.002*	0.28 (0.04–1.78) 0.176	0.58 (0.15–2.31) 0.440
D6 < 6 ≥ 6 P	0.70 (0.30–1.65) 0.416	1.05 (0.79–1.40) 0.749	1.34 (0.77–2.34) 0.295	0.98 (0.42–2.28) 0.964	0.59 (0.19–1.80) 0.350

Characteristics	Excess weight*	Energy	Protein	Vegetable	Fibers	Fruit
	PR (CI95%)	PR (CI95%)	PR (CI95%)	PR (CI95%)	PR (CI95%)	PR (CI95%)
D1 < 8 ≥ 8*P*	0.98 (0.54–1.80) 0.957	1.07 (0.94–1.21) 0.329	0.26 (0.06–1.20) 0.085	1.02 (0.75–1.38) 0.910	0.86 (0.67–1.09) 0.203	0.98 (0.71–1.36) 0.907
D2 < 6 ≥ 6*P*	1.94 (1.08–3.51) 0.028*	1.05 (0.90–1.22) 0.560	0.99 (0.37–2.66) 0.991	0.61 (0.46–0.81) 0.001*	0.84 (0.68–1.04) 0.102	0.88 (0.68–1.14) 0.331
D3 < 10 ≥ 10*P*	0.96 (0.55–1.67) 0.873	1.04 (0.86–1.26) 0.693	1.39 (0.38–5.10) 0.622	1.02 (0.76–1.36) 0.899	1.01 (0.84–1.21) 0.946	0.94 (0.70–1.27) 0.690
D5 < 12 ≥ 12*P*	0.68 (0.37–1.25) 0.213	1.14 (1.02–1.29) 0.025*	1.30 (0.34–4.96) 0.703	0.84 (0.53–1.35) 0.479	1.42 (1.15–1.75) 0.001*	1.06 (0.74–1.51) 0.739
D6 < 6 ≥ 6*P*	1.0 (0.53–1.88) 0.994	0.92 (0.76–1.12) 0.422	0.68 (0.21–2.23) 0.523	0.96 (0.72–1.28) 0.795	0.77 (0.59–1.02) 0.068	0.880 (0.63–1.23) 0.453

Problematic behaviors at mealtimes were accessed by the Dimensions of the Eating Behavior Assessment Scale in Autism Spectrum Disorder (28). Average scores of the eating behavior scale dimensions (D) (28): D1 – Masticatory motor skills; D2 – Food selectivity; D3 – Mealtime skills; D5 – Rigid eating behaviors; D6 – Oppositional eating behavior. Dimensions 4 (“Inappropriate mealtime behaviors”) and 7 (“Food allergies and intolerance”) were omitted from the analysis due to their low occurrence. 95%CI: 95% confidence interval. Adjustment variables: Sex, age, skin color, maternal profession, economic class, medications. *Statistical significance (*p* < 0.05).

## Discussion

The present study analyzed the association between problematic mealtime behaviors and components of food consumption and nutritional status in children with ASD. Our study demonstrated the occurrence of problematic behavior at mealtimes in all children studied, with a predominance of food selectivity. Furthermore, analysis of Poisson regression with robust variance confirmed that food selectivity and rigid eating behaviors impacted distinctly food consumption, regardless confounders. Our results also pointed to a possible influence of the refusal of vegetables in children with ASD and the occurrence of excess body weight.

In our study, food selectivity was present in more than a half of children, corroborating with the literature (10). Food selectivity rates among children with ASD range from 13 to 87% (14, 15). When children with similar ages of our study were accessed (2–10 years), a rate of 84.8% was observed (8) and, in Brazilian children with a median age of 10.5 years, it was 31.9% (29). This variation may be due to differences in the age ranges of study participants, as the frequency of selectivity may decrease with age (9), and also due to the fact that studies used different methodologies and definitions of food selectivity (4, 15), thus making data homogeneity difficult.

Food selectivity is the most studied problematic behavior in ASD (14, 15). However, given the different methodologies and definitions for its assessment (15), the parameters used may include behaviors characteristic of rigid eating. For example, during identification of food selectivity, studies included the percentage of food refusal (4, 12), restricted food variety (30) persistence in routine (31), and food refusal, limited food repertoire, and high frequency of single food intake (9). Nevertheless, our findings demonstrated that food selectivity was associated with no vegetable consumption, while rigid eating behavior was positively associated with inadequate calorie intake and lower intake of fiber, calcium and zinc. These results highlight the need to distinguish food selectivity of rigid eating behaviors when exploring children’s eating and nutritional parameters since, as verified, their implications on food consumption and nutritional status do not seem to coincide.

Higher scores of food selectivity were associated with no vegetable consumption. Previous studies have described the low consumption of fruits and vegetables as a dietary characteristic of people with ASD (11, 12). A study that investigated the food selectivity of preschool and school-aged children showed low daily consumption of fruits and vegetables (32). Another study (12) also demonstrated that children with high food selectivity scores consumed a significantly smaller variety of fruits and vegetables compared to the control group. Sensory sensibility to food texture has been identified as an important condition for food selectivity (10, 11) and the refusal of vegetables and fruits (33). In our sample, we did not observe an association between food selectivity and low fruit consumption, which may indicate that food selectivity could affects less the tolerance for fruits than for vegetables in children with ASD (33).

Regarding rigid eating behavior, to the best of our knowledge, our study is the first to investigate its relationship with food consumption. In ASD, low consumption of fiber (4, 33) and zinc (31) is recorded, while regarding calories, previous study reports association with low—and not with excessive—consumption (34). In fact, we verified low consumption of fiber and zinc in children with high rigidity but, regarding calorie intake, excess and lack of calories coexisted in this group. For calcium, studies on food consumption in ASD more commonly indicate inadequacy in its consumption (4, 31, 33) than adequacy (34). We found that, among children with high rigidity, there was a lower frequency of calcium inadequacy. This was due to the consumption of dairy drinks (milk, yogurt, chocolate milk), present at breakfast, snacks, and large meals. Although rigid eating behavior favored the nutritional status of calcium, these children’s diet expressed monotony and not necessarily indicated better nutritional pattern. During the 3 days of dietary recall, we observed repetitive food consumption as well as no more than three types of food at large meals, which were commonly accompanied by the consumption of dairy drinks. From this, knowing that in ASD there is a higher frequency of milk consumption (10, 34) but that this is accompanied by food monotony and replaced of large meals by dairy products, the influence of rigid eating behavior on calcium status needs to be interpreted cautiously.

When evaluating the nutritional status of children with ASD, excessive body weight was observed in more than 40% of children, with similar ratings of overweight and obesity. These results coincide with the literature that indicates rates of 10.9–26.2% for overweight (8, 31, 32) and 16.3–32.3% for obesity (4, 8, 31, 32). Excessive body weight is a worldwide problem for children in general due to the risk of obesity in adulthood (35) and it is worrying to observe that, among children with ASD, the frequencies of overweight and obesity are higher than in children without ASD (7). Interestingly, our results demonstrated that food selectivity was associated with excess body weight. Previous studies showed divergent results regarding the presence of excess body weight in selective children with ASD, with two studies supporting our findings (4, 31). A study (31) showed the presence of overweight or obesity in almost 60% of individuals with ASD aged 4–18 years, whose main eating problem was food selectivity. The other (4), conducted with subjects of 6 to 18 years old, identified that higher food selectivity scores was accompanied by both low weight (18.4% ASD vs. 3.20% control) and obesity (16.3% ASD vs. 8.6% control). However, two studies (8, 33) observed no significant association between food selectivity and obesity. Considering that the refusal of vegetables and fruits is the characteristic consensually related to food selectivity (14), we suggest further studies to confirm whether excess body weight in selective children with ASD (4, 31) could be attributed to the refusal of vegetables and fruits.

Our study provided evidence for the use of the Eating Behavior Assessment Scale for ASD (28) as a valuable tool in investigations into problematic mealtime behaviors, as it allows the distinct identification of mealtime behaviors which potential to impact food consumption and nutritional status. The advantages of this study were to provide more data on consumption and eating behavior of children with ASD in Brazil, where we still have few studies, but an exponentially growing prevalence. Also, we highlight as a favorable point of the study the fact that the researcher has long experience in nutritional guidance in an institution that cares for children with ASD, which provided quality to the data obtained. Despite the relatively large sample size when compared to studies involving children with ASD, it could be considered as limitations the fact this is a single center study and that control group was not included, which could which compromises the inference of the results. Also, the lack of specific instruments for addressing food consumption and measuring the nutritional status of children with ASD are limitations to consider. Because the interplay between problematic behaviors at mealtimes, food consumption, and nutritional status in ASD is complex and poorly explored by scientific literature, we suggest the conduction of longitudinal studies to identify the temporal sequence and causal pathways to these relationships and whether these pathways are modulated or diverge across typical and atypical development in ASD. Nevertheless, our results support the investigation of problematic behaviors at mealtimes during nutritional therapy for children with ASD.

## Conclusion

Food selectivity and rigid eating behaviors have different relationships with food consumption and are associated with a higher prevalence of dietary inadequacies, which can lead to nutritional risks for the child with ASD. The food selectivity, given by the refusal of vegetables, was associated with excess body weight. This study highlighted the importance of investigating problematic mealtime behaviors to provide more assertive nutritional interventions for these individuals. This study demonstrated that selective eaters consume fewer vegetables, and that identifying rigidity is important due to its relationship with the studied nutrients. This information can guide more effective nutritional interventions and support the development of personalized strategies for managing problematic mealtime behaviors in ASD.

## Data Availability

The original contributions presented in the study are included in the article/Supplementary material, further inquiries can be directed to the corresponding authors.
