# Supplementary material for: Problematic behaviors at mealtimes and the nutritional status of Brazilian children with Autism Spectrum Disorder
Source: Front Public Health. 2024 Oct 14;12:1392478. doi: 10.3389/fpubh.2024.1392478 (PMC11513654; doi:10.3389/fpubh.2024.1392478)
Supplement: Supplementary file 1 [file Table_1.docx]

Supplementary Table - Frequency of Children's Weight, Fiber and Nutrient Variables According to Eating Behavior Scale Items, in Brazilian children (2 to 10 years) with autism spectrum disorder (n = 90), Viçosa, Brazil (2022)

|  | Variables | BMI/Age | | P | Total Calories | | P | Protein | | P | Fiber | | P |
| --- | --- | --- | --- | --- | --- | --- | --- | --- | --- | --- | --- | --- | --- |
|  |  | With excess weight | Without excess weight |  | Adequate | Inadequate |  | Adequate | Inadequate |  | Adequate | Inadequate |  |
| D1 | ≥ 8 | 9 (39,1) | 14 (60,9) | 0,823 | 1 (4,3) | 22 (95,7) | 0,438 | 22 (95,7)) | 1 (4,3) | 0,277 | 5 (21,7) | 18 (78,3) | 0,449 |
|  | <8 | 28 (41,8) | 39 (58,2) |  | 8 (11,9) | 59 (88,1) |  | 57 (85,1) | 10 (14,9 |  | 10 (14,9) | 57 (85,1) |  |
| D2 | ≥ 6 | 26 (50,0) | 26 (50,0) | **0,045** | 4 (7,7) | 48 (92,3) | 0,485 | 45 (86,5) | 7 (13,5) | 0,754 | 9 (17,3) | 43 (82,7) | 0,849 |
|  | < 6 | 11 (28,9) | 27 (71,1) |  | 5 (13,2) | 33 (86,8) |  | 34 (89,5) | 4 (10,5) |  | 6 (15,8) | 32 (84,2) |  |
| D3 | ≥ 10 | 18 (58,1) | 13 (41,9) | 0,908 | 3 (9,7) | 28 (90,3) | 1,000 | 27 (87,1) | 4 (12,9) | 1,000 | 6 (19,4) | 25 (80,6) | 0,620 |
|  | < 10 | 35 (59,3) | 24 (40,7) |  | 6 (10,2) | 53 (89,9) |  | 52 (88,1) | 7 (11,9) |  | 9 (15,3) | 50 (84,7) |  |
| D4 | ≥ 4 | 1 (50,0) | 1 (50,0) | 1,000 | 0 (0,0) | 2 (100,0) | 1,000 | 2 (100,0) | 0 (0,0) | 1,000 | 1 (50,0) | 1 (50,0) | 0,307 |
|  | < 4 | 52 (59,1) | 36 (40,9) |  | 9 (10,2) | 79 (89,8) |  | 77 (87,5) | 11 (12,5) |  | 14 (15,9) | 74 (84,1) |  |
| D5 | ≥ 12 | 11 (61,1) | 7 (38,9) | 0,830 | 0 (0,0) | 18 (100,0) | 0,195 | 16 (88,9) | 2 (11,1) | 1,000 | 0 (0,0) | 18 (100,0) | **0,035** |
|  | < 12 | 42 (58,3) | 30 (41,7) |  | 9 (12,5) | 63 (87,5) |  | 63 (87,5) | 9 (12,5) |  | 15 (20,8) | 57 (79,2) |  |
| D6 | ≥ 6 | 14 (63,6) | 8 (36,4) | 0,603 | 3 (13,6) | 19 (86,4) | 0,683 | 20 (90,9) | 2 (9,1) | 1,000 | 6 (27,3) | 16 (72,7) | 0,125 |
|  | < 6 | 39 (57,4) | 29 (42,6) |  | 6 (8,8) | 62 (91,2) |  | 59 (86,8) | 9 (13,2) |  | 9 (13,2) | 59 (86,8) |  |
| D7 | ≥ 6 | 3 (100,0) | 0 (0,0) | 0,266 | 0 (0,0) | 3 (100,0) | 1,000 | 3 (100,0) | 0 (0,0) | 1,000 | 0 (0,0) | 3 (100,0) | 1,000 |
|  | < 6 | 50 (57,5) | 37 (42,5) |  | 9 (10,3) | 78 (89,7) |  | 76 (87,4) | 11 (12,6) |  | 15 (17,2) | 72 (82,8) |  |

Notes: Total calorie, protein, iron, calcium, zinc, and fiber intakes were assessed according to the Dietary Reference Intakes^27^. Mean scores of the eating behavior scale factors were as follows: D1: Chewing Motor Skills; D2: Food Selectivity; D3: Mealtime Skills; D4: Inappropriate Mealtime Behavior; D5: Rigid Eating Behaviors; D6: Oppositional Eating Behavior; and D7: Food Allergies and Intolerances^18^. Pearson chi-square test and Fisher's exact test were used *Statistical significance (p < 0.05).

Supplementary Table - Frequency of Variables for Children's Weight, Fiber, and Nutrients According to the Items of the Labirinto Scale, Viçosa – MG (2022)

|  | Variables | Iron | | P | Calcium | | P | Zinc | | P | Fruit Consumption | | P | Veg./Leg. Consumption | | P |
| --- | --- | --- | --- | --- | --- | --- | --- | --- | --- | --- | --- | --- | --- | --- | --- | --- |
|  |  | Adequate | Inadequate |  | Adequate | Inadequate |  | Adequate | Inadequate |  | Present | Absent |  | Present | Absent |  |
| D1 | ≥ 8 | 19 (82,6) | 4 (17,4) | 0,144 | 10 (43,5) | 13 (56,5) | 0,232 | 12 (52,2) | 11 (47,8) | 0,528 | 16 (69,6) | 7 (30,4) | 0,742 | 15 (65,2) | 8 (34,8) | 0,659 |
|  | <8 | 45 (67,2) | 22 (32,8) |  | 20 (29,9) | 47 (70,1) |  | 40 (59,7) | 27 (40,3) |  | 49 (73,1) | 18 (26,9) |  | 47 (70,1) | 20 (29,9) |  |
| D2 | ≥ 6 | 38 (73,1) | 14 (26,9) | 0,630 | 18 (34,6) | 34 (65,4) | 0,763 | 28 (53,8) | 24 (46,2) | 0,377 | 34 (65,4) | 18 (34,6) | 0,090 | 27 (51,9) | 25 (48,1) | **<0,001** |
|  | < 6 | 26 (68,4) | 12 (31,6) |  | 12 (31,6) | 26(68,4) |  | 24 (63,2) | 14 (36,8) |  | 31 (81,6) | 7 (18,4) |  | 35 (92,1) | 3 (7,9) |  |
| D3 | ≥ 10 | 23 (74,2) | 8 (25,8) | 0,640 | 10 (32,3) | 21 (67,7) | 0,875 | 17 (54,8) | 14 (45,2) | 0,682 | 21 (67,7) | 10 (32,3) | 0,492 | 20 (64,5) | 11 (35,5) | 0,516 |
|  | < 10 | 41(69,5) | 18 (30,5) |  | 20 (33,9) | 39 (66,1) |  | 35 (59,3) | 24 (40,7) |  | 44 (74,6) | 15 (25,4) |  | 42 (71,2) | 17 (28,8) |  |
| D4 | ≥ 4 | 1 (50,0) | 1 (50,0) | 0,497 | 1 (50,0) | 1 (50,0) | 1,000 | 2 (100,0) | 0 (0,0) | 0,507 | 2 (100,0) | 0 (0,0) | 1,000 | 1 (50,0) | 1 (50,0) | 0,528 |
|  | < 4 | 63 (71,6) | 25 (28,4) |  | 29 (33,0) | 59 (67,0) |  | 50 (56,8) | 38 (43,2) |  | 63 (71,6) | 25 (28,4) |  | 61 (69,3) | 27 (30,7) |  |
| D5 | ≥ 12 | 12 (66,7) | 6 (33,3) | 0,642 | 11 (61,1) | 7 (38,9) | **0,005** | 4 (22,2) | 14 (77,8) | **0,001** | 12 (66,7) | 6 (33,3) | 0,556 | 9 (50,0) | 9 (50,0) | 0,053 |
|  | < 12 | 52 (72,2) | 20 (27,8) |  | 19 (26,4) | 53 (73,6) |  | 48 (66,7) | 24 (33,3) |  | 53 (73,6) | 19 (26,4) |  | 53 (73,6) | 19 (26,4) |  |
| D6 | ≥ 6 | 17 (77,3) | 5 (22,7) | 0,463 | 7 (31,8) | 15(68,2) | 0,862 | 10 (45,5) | 12 (54,5) | 0,178 | 15 (68,2) | 7 (31,8) | 0,626 | 16 (72,7) | 6 (27,3) | 0,655 |
|  | < 6 | 47 (69,1) | 21 (30,9) |  | 23 (33,8) | 45 (66,2) |  | 42 (61,8) | 26 (38,2) |  | 50 (73,5) | 18 (26,5) |  | 46 (67,6) | 22 (32,4) |  |
| D7 | ≥ 6 | 3 (100,0) | 0 (0,0) | 0,554 | 2 (66,7) | 1 (33,3) | 0,257 | 2 (66,7) | 1 (33,3) | 1,000 | 2 (66,7) | 1 (33,3) | 1,000 | 1 (33,3) | 2 (66,7) | 0,227 |
|  | < 6 | 61 (70,1) | 26 (29,9) |  | 28 (32,2) | 59 (67,8) |  | 50 (57,5) | 37 (42,5) |  | 63 (72,4) | 24 (27,6) |  | 61 (70,1) | 26 (29,9) |  |

Notes: Total calorie, protein, iron, calcium, zinc, and fiber intakes were assessed according to the Dietary Reference Intakes^27^. Mean scores of the eating behavior scale factors were as follows: Factor 1: Chewing Motor Skills; Factor 2: Food Selectivity; Factor 3: Mealtime Skills; Factor 4: Inappropriate Mealtime Behavior; Factor 5: Rigid Eating Behaviors; Factor 6: Oppositional Eating Behavior; and Factor 7: Food Allergies and Intolerances^18^. Pearson chi-square test and Fisher's exact test were used *Statistical significance (p < 0.05).
